# Inhibitors of Cyclic Dinucleotide Phosphodiesterases and Cyclic Oligonucleotide Ring Nucleases as Potential Drugs for Various Diseases

**DOI:** 10.3390/cells14090663

**Published:** 2025-04-30

**Authors:** Christopher S. Vennard, Samson Marvellous Oladeji, Herman O. Sintim

**Affiliations:** 1 Chemistry Department, Purdue University, West Lafayette, IN 47907, USA; cvennard@nd.edu (C.S.V.); soladeji@nd.edu (S.M.O.); 2 Department of Chemistry and Biochemistry, University of Notre Dame, Notre Dame, IN 46556, USA

**Keywords:** cyclic dinucleotides, cyclic oligonucleotides, phosphodiesterase

## Abstract

The phosphodiester linkage is found in DNA, RNA and many signaling molecules, such as cyclic mononucleotide, cyclic dinucleotides (CDNs) and cyclic oligonucleotides (cONs). Enzymes that cleave the phosphodiester linkage (nucleases and phosphodiesterases) play important roles in cell persistence and fitness and have therefore become targets for various diseased states. While various inhibitors have been developed for nucleases and cyclic mononucleotide phosphodiesterases, and some have become clinical successes, there is a paucity of inhibitors of the recently discovered phosphodiesterases or ring nucleases that cleave CDNs and cONs. Inhibitors of bacterial c-di-GMP or c-di-AMP phosphodiesterases have the potential to be used as anti-virulence compounds, while compounds that inhibit the degradation of 3′,3′-cGAMP, cA_3_, cA_4_, cA_6_ could serve as antibiotic adjuvants as the accumulation of these second messengers leads to bacterial abortive infection. In humans, 2′3′-cGAMP plays critical roles in antiviral and antitumor responses. ENPP1 (the 2′3′-cGAMP phosphodiesterase) or virally encoded cyclic dinucleotide phosphodiesterases, such as poxin, however, blunt this response. Inhibitors of ENPP1 or poxin-like enzymes have the potential to be used as anticancer and antiviral agents, respectively. This review summarizes efforts made towards the discovery and development of compounds that inhibit CDN phosphodiesterases and cON ring nucleases.

## 1. Cyclic Oligonucleotides as Signaling Molecules

**Figure 1:**
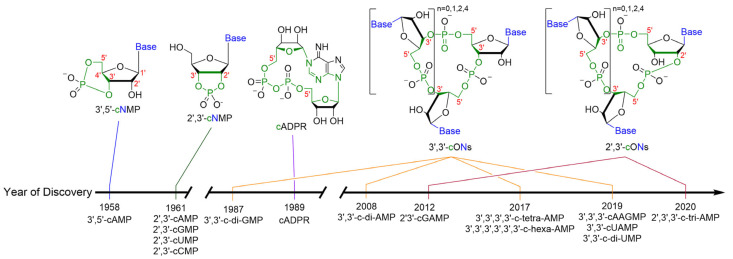
The years of discovery for cyclic nucleotide phosphates, including 3′,5′-cAMP [1,2], 2′,3′-cAMP [18], 3′,3′-c-di-GMP [10], cADPR [19], c-di-AMP [20], 2′,3′-cGAMP [13], c-tetra-AMP (cA_4_) and c-hexa-AMP (cA_6_) [21,22], 3′,3′,3′-cAAGMP [23], 3′,3′-cUAMP [23], 3′3′-c-di-UMP [23], and 2′,3′,3′,-c-tri-AMP [24].

Environmental signals and intracellular fitness must be constantly monitored for individual cells to survive. This fundamental principle of fitness holds true across the entire tree of life. A common signaling motif used for this purpose is the utilization of second messenger molecules. A receptor will be activated by a stimulus and release a second messenger. This second messenger will physically travel and bind to another receptor to elicit a response. Nucleotide-based second messengers, both cyclic and linear forms, play important roles in signal transduction. Earl Sutherland ushered in the golden era of cyclic mononucleotide research with his seminal reports on cyclic adenylate mononucleotide (cAMP) [1,2]. In the ensuing years following Sutherland’s groundbreaking work, it was revealed that cAMP and cGMP played critical roles in many processes, leading to the development of drugs that targeted cAMP and cGMP-related phosphodiesterases (PDEs) for the treatment of diverse diseases, such as erectile dysfunction [3], respiratory diseases [4], cardiovascular [5,6] and other diseases. This review is focused on cyclic di- and oligo-nucleotides, but those interested in inhibitors of cyclic mononucleotide PDEs are directed to some recent reviews on cAMP and cGMP PDEs [7,8,9]. In the late 1980s, Benziman published a series of papers that revealed the structure and function of c-di-GMP, an archetypical cyclic dinucleotide (CDN) [10,11]. Since Benziman’s original work, other types of cyclic dinucleotides with 3′-5′ phosphodiester linkages (for example c-di-AMP, cGAMP) were discovered in bacteria. In bacteria, the role of cyclic oligonucleotides (cONs) as second messenger molecules has been rapidly expanding since their discovery [10,11,12]. In higher organisms, cyclic dinucleotides that contain a non-canonical 2′3′-phosphodiester linkage have been described [13,14,15,16]. In humans, the inflammatory response caused by CDNs has been gaining attention over the last decade as specific druggable targets have emerged [17]. A timeline of cON discovery is shown in Figure 1. For the purpose of this review, CDNs will also be classified as cONs. The biologically relevant cONs described thus far have between 2 and 6 nucleobases.

**Figure 2:**
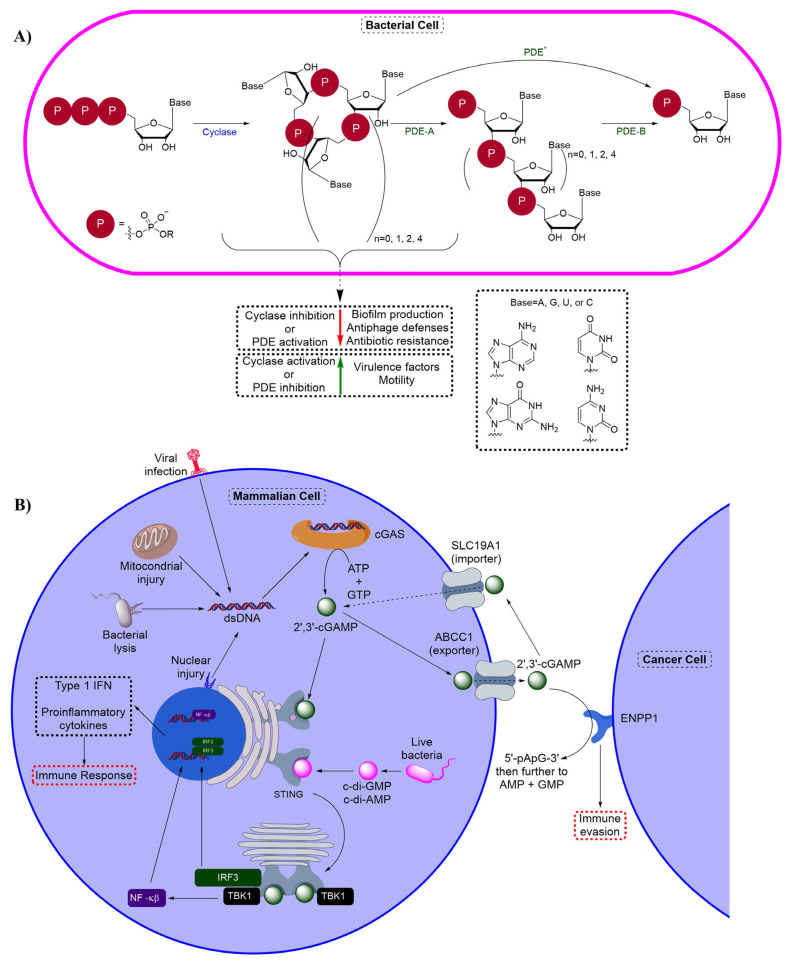
(**A**) Bacterial cON signaling overview showing the synthesis (cyclase) and degradation (PDE) of bacterial cONs. (**B**) Mammalian cON overview showing the synthesis of 2′,3′-cGAMP by cGAS, sensing by STING, and degradation by ENPP1.

Since cONs are signal activators, the concentrations of these second messenger molecules are highly regulated in cells. cONs are enzymatically synthesized from nucleotide triphosphates (NTPs) by cyclases, degraded by phosphodiesterases to linear oligonucleotides and then further degraded into nucleotide monophosphates (NMPs) (Figure 2). Interestingly, some isoforms of PDEs can hydrolyze cONs all the way to NMPs, whereas other isoforms are only able to cleave one phosphodiester bond. Isoforms of PDEs that only cleave one phosphodiester bond require the aid of other hydrolase enzymes to further process the linear oligonucleotides to NMPs. Specifically, oligoribonuclease (Orn) and other nano-RNase (NrnA) enzymes are responsible for this process.

Although there are both linear [25] and cyclic nucleotide phosphates (CNPs) that operate as signaling molecules, the cyclic nucleotides are much more diverse in their isomerism and function. 3′,5′-cAMP and 3′,5′-cGMP are among the most widely studied, as these two CNPs are responsible for smooth muscle relaxation, bronchodilation, and vasodilation in humans [26,27,28]. In bacteria, 3′,5′-cAMP and 3′,5′-cGMP have been linked to a myriad of processes through gene expression regulation [12,29]. In addition to the canonical 3′-5′ phosphate linkage, 2′3′-CNPs have also recently been found to bind to bacterial ribosomes, inhibiting gene expression at a translational level [30,31].

**Figure 3:**
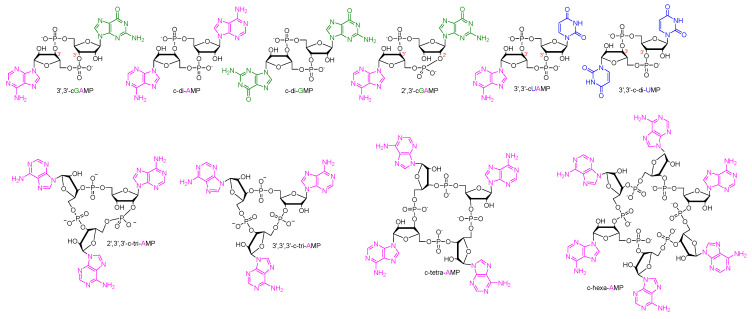
Structures of notable cONs which are discussed in detail in this review.

The dinucleotides are even more complex than their mononucleotide counterparts, as the nucleotide bases may either be homologous or heterologous in addition to utilizing various linkage patterns (Figure 3). The current understanding is that bacteria most commonly produce cONs with a 3′,3′-linkage (c-di-AMP, c-di-GMP, cUAMP, cGAMP, cGAAMP, cA_4_, and cA_6_) (Figure 2A) whereas eukaryotic cells produce 2′,3′-cGAMP. However, eukaryotic cells also identify c-di-AMP and c-di-GMP as a way of monitoring pathogens via the STING pathway (Figure 2B) [32]. Even though we have a strong understanding of the biological roles of these messengers after decades of research, modulation of cON signaling pathways is still underutilized in drug discovery. Historically, cyclase enzymes have been targeted with small-molecule inhibitors. However, recently, appreciation has increased for the roles of cONs in disease states and efforts are increasing to discover novel cON PDE inhibitors.

## 2. Bacterial cON PDEs as Potential Drug Targets

### 2.1. Targeting Bacterial Cyclic-di-GMP PDEs

Although cyclic di-GMP (c-di-GMP) was initially identified in 1987 as an allosteric activator of cellulose synthase [10], its broader role as a ubiquitous bacterial second messenger became evident in the 21st century. This signaling molecule is now recognized for its involvement in biofilm formation, flagella regulation, and virulence modulation, as well as its influence on the bacterial cell cycle and development [33,34,35,36]. c-di-GMP is synthesized by the GGDEF domain proteins utilizing 2 molecules of GTP. Then, degradation can take place using EAL or HD-GYP domains proteins with c-di-GMP specific phosphodiesterase activities. GGDEF domains catalyze the condensation of two molecules of GTP to generate c-di-GMP, while EAL or the HD-GYP domains are responsible for c-di-GMP hydrolysis to the linear molecule pGpG. There are some reports of HD-GYP domains hydrolyzing the second phosphodiester bind to result in GMP [37]. For the enzymes that cannot fully hydrolyze c-di-GMP to GMP, enzymes such as oligoribonuclease are utilized to further degrade pGpG to GMP [38].

### 2.2. EAL PDEs

**Figure 4:**
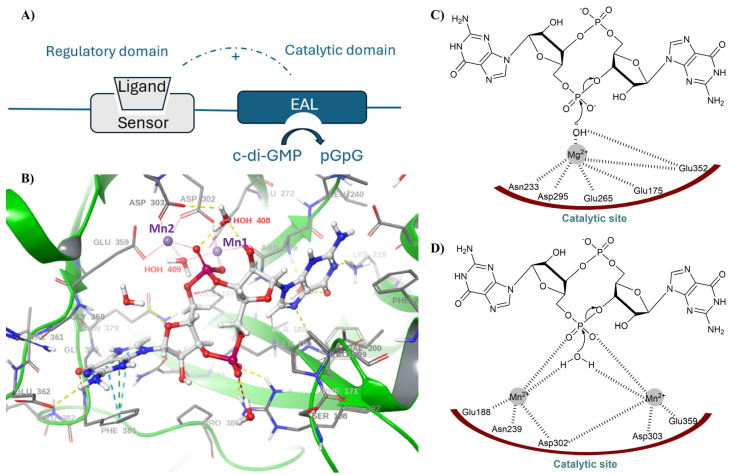
(**A**) A typical EAL PDE catalytic domain with a regulatory domain [39,40,41,42,43]. (**B**) Proposed Mg^2+^-assisted, general base-catalyzed hydrolysis of c-di-GMP [46]. (**C**) The crystal structure of BlrP1 (PDB: 3GG0) reveals Mn^2+^ ions (shown in purple) coordinated by multiple carboxylate residues. Among these, Glu359, positioned on strand β7EAL, plays a key role, along with Asp302 and Asp303, which are located on a loop near the dimer interface. Additionally, the structure highlights a potential hydrolytic solvent molecule, HOH409, situated in a possible near-attack conformation, as well as HOH408, a water molecule that may function as a proton donor for the leaving group [45]. (**D**) Proposed two-metal mediated catalytic mechanism of c-di-GMP hydrolysis [47,48].

EAL PDEs are widespread in bacteria and are often associated with sensory domains such as GGDEF (modified), PAS, REC, or GAF, which allow them to respond to environmental signals (Figure 4A) [39,40,41,42,43]. Interestingly, some EAL domain proteins, such as FimX from *Pseudomonas aeruginosa*, are catalytically inactive and instead function as high-affinity c-di-GMP receptors [44]. The regulation of EAL domain activity is further influenced by environmental factors such as pH, metal ion concentration, and even light in the case of photoreceptor proteins like BlrP1 [45].

The structural characterization of EAL domain proteins has provided significant insights into their function. Crystal structures of proteins such as BlrP1 from *Klebsiella pneumoniae* and TBD1265 from *Thiobacillus denitrificans* have revealed that EAL domains adopt a variation in the classic (αβ)8 TIM barrel fold [45,49]. The active site is located at the C-terminal end of the barrel and is usually occupied by two divalent metal ions when bound to c-di-GMP.

Liang and co-workers first proposed the catalytic mechanism of EAL PDEs, using RocR from *P. aeruginosa* and tdEAL from *T. denitrificans* [46]. They described a Mg^2+^-assisted, general base-catalyzed hydrolysis of c-di-GMP, where a conserved Glu in the EAL motif acts as a base catalyst, deprotonating a Mg^2+^-coordinated water molecule to generate a nucleophilic hydroxide ion for cleavage (Figure 4B). Later, the crystal structure of BLUF-EAL PDE Blrp1 from *Klebsiella pneumoniae* in complex with its substrate led to the proposal of a two-metal ion mechanism. In this model, two metal ions are coordinated by essential carboxylate residues, including Glu 359 from the EAL motif, a bridging water molecule, and the phosphate oxygen of c-di-GMP (Figure 4C,D). Subsequently, a general two-metal-ion mechanism was proposed for EAL domain enzymes, similar to other nucleotide phosphodiesterases, where one metal ion activates a water molecule for nucleophilic attack, while the other stabilizes the transition state and facilitates leaving group formation [47,48].

**Figure 5:**
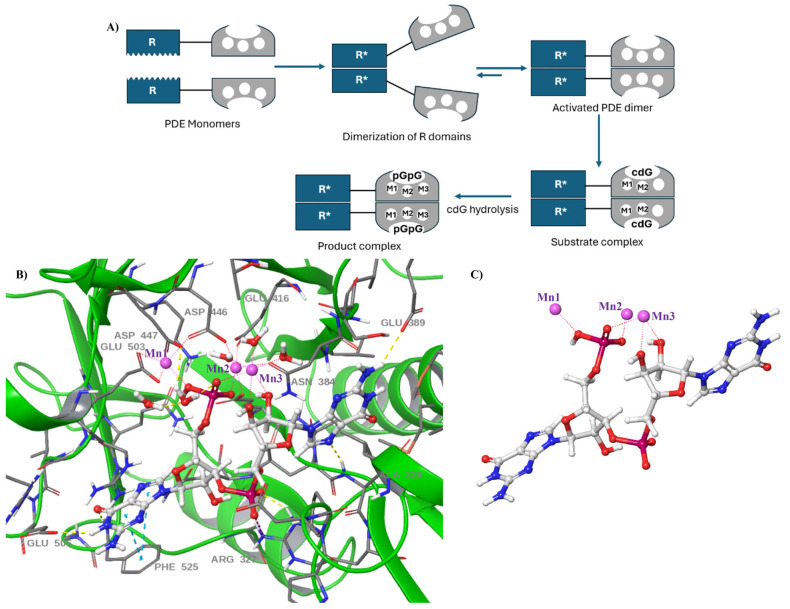
(**A**) A proposed dimerization mechanism regulating c-di-GMP hydrolysis catalyzed by EAL-domain PDE. A full-length EAL PDE, equipped with a regulatory (R) domain, undergoes a structural transition upon signal detection. The monomeric protein dimerizes through interactions between its R domains, which in turn facilitates the dimerization of the EAL domains, leading to the formation of an active PDE dimer. Once activated, this PDE dimer coordinates metal ions at the M1 and M2 sites via conserved aspartate residues, enabling substrate binding. A third metal ion occupies the M3 site, playing a crucial role in stabilizing the negatively charged transition state during the hydrolysis of c-di-GMP into pGpG [39,40,41,42,43]. (**B**) Crystal structure of CC3396^EAL^ (PDB code: 3U2E) with manganese ions (purple) occupying M1, M2 and M3 sites [53]. (**C**) pGpG ligand structure showing the interactions with the coordinated metal ions [53].

Many EAL proteins function as dimers, and dimerization appears to be a crucial requirement for enzymatic activity [50]. Using biophysical and functional analyses, Schirmer and co-workers showed that the isolated PDE EAL domain from *E. coli* (YahA) is in a fast thermodynamic equilibrium between a monomer and dimer, and that the EAL domain is only active in its dimeric form [51]. The rate of dimerization is shown to be increased by about 100-fold upon substrate binding, and specific site-directed mutagenesis preventing dimerization results in reduced PDE activity without changing the substrate binding affinity. This finding was corroborated by Dom et al., whose recent structural analyses of EAL domains from *Pseudomonas aeruginosa* PA1727 (MucR^EAL^) and PA3825 (PA3825^EAL^) revealed that EAL PDE activity is modulated by different dimerization interfaces [50]. This dimerization is driven by variations in the helix α5 length and the precise positioning of the metal-coordinating motif DDFGTG, which facilitates the formation of the active site. The structural transitions that differentiate the substrate-bound and product-bound states of EAL phosphodiesterases (EAL-PDEs) are illustrated schematically in Figure 5A.

Furthermore, for the first time, crystal structure of PA3825^EAL^ bound to hydrolysis product, pGpG, suggested that more than two metal ions may be required for EAL-catalyzed hydrolysis of c-di-GMP where manganese and sodium ions occupy M1 and M3 sites, respectively [50]. The pGpG complex revealed a novel metal-binding site where pGpG directly coordinates a metal ion through its phosphate oxygens and ribosyl-2′-O leaving group. This site was further validated by the EAL domain of *Caulobacter crescentus* CC3396 (CC3396^EAL^), which also exhibited a third metal-binding site (M3), in addition to the well-characterized M1 and M2 sites (Figure 5B,C) [50]. The presence of M3 near the hydrolyzed phosphodiester bond led to the hypothesis that it stabilizes the negatively charged transition state during c-di-GMP hydrolysis, supported by the involvement of pGpG’s non-bridging phosphate oxygens in metal coordination. This discovery parallels findings in HD-GYP domain PDEs, which also appear to utilize three-metal-ion mechanisms. Furthermore, comparisons with endonucleases suggest that three metal ions may be a general feature of nucleotide hydrolysis enzymes [52].

### 2.3. HD-GYP PDEs

HD-GYP phosphodiesterases (PDEs) are metal-dependent enzymes that belong to the HD phosphohydrolase superfamily but contain an additional GYP motif and are distinct from EAL-domain PDEs [54]. HD-GYP PDEs are assumed to be less widespread than EAL PDE and are often mutually exclusive with EAL-domain PDEs. Unlike EAL-domain PDEs, which hydrolyze c-di-GMP into linear pGpG, HD-GYPs can further degrade pGpG into GMP. The HD domain is characterized by a conserved catalytic dyad of histidine (H) and aspartate (D), which is necessary for coordinating divalent metal ions. Bioinformatics analysis shows that HD-GYP domains are prevalent in bacterial genomes, particularly in Proteobacteria and Firmicutes, but absent in archaea and eukaryotes [55]. The first identified HD-GYP PDE, RpfG from *Xanthomonas campestris*, is well characterized for its role in c-di-GMP hydrolysis [56]. It is linked to a two-component regulatory system, where the sensor histidine kinase RpfC phosphorylates RpfG upon detecting quorum-sensing signals, activating its PDE function. This activation reduces c-di-GMP levels, leading to the induction of virulence genes and inhibition of biofilm formation. Other bacterial species also exhibit HD-GYP-mediated regulation of biofilm formation and motility such as *Vibrio cholerae*, *Borrelia burgdorferi*, and *Pseudomonas aeruginosa* [57,58,59].

The HD-GYP domain adopts an all-α fold with a catalytic metal-binding core. The GYP loop, which extends beyond the conserved HD motif, plays a role in substrate binding and possibly protein–protein interactions [59]. HD-GYP PDEs also form dimers, and dimerization may regulate enzymatic activity [60]. The PDE activity of HD-GYP proteins depends on the presence of metal ions, typically Mg^2+^, Mn^2+^, or Fe^2+^, with some enzymes requiring a reduced di-iron center for function [61].

**Figure 6:**
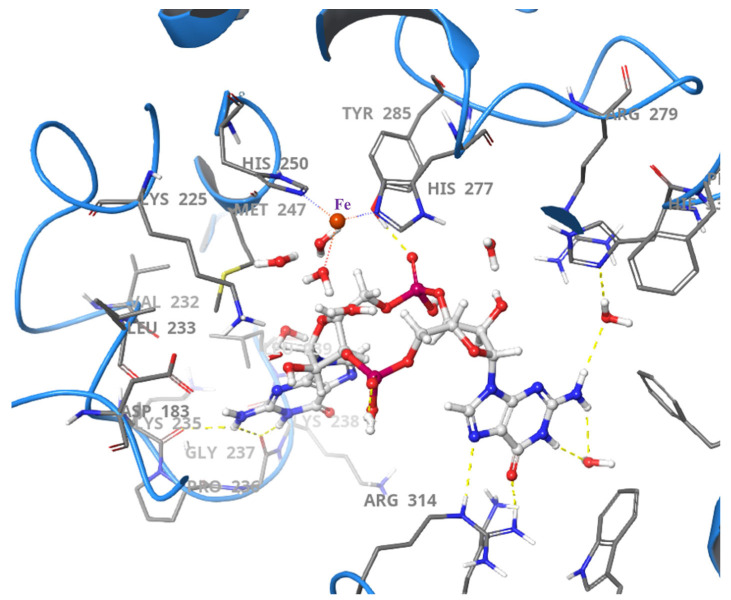
Crystal structure of PmGH (4MDZ) shows the interaction between the c-di-GMP and the essential active site residues [62].

In *Pseudomonas marina* PDE (PmGH), iron ions interact with metal-binding residues to stabilize the oxygen atoms within the central 12-membered ribose-phosphate ring of c-di-GMP. The Tyr285 residue, a key component of the GYP motif, plays a crucial role in strengthening binding and improving substrate specificity by forming a hydrogen bond with one of the nonbridging oxygen atoms in the ribose-phosphate ring (Figure 6). The presence of a third metal-binding residue, such as Glu185 in PmGH, determines whether the enzyme forms a trinuclear or binuclear metal center, influencing its ability to hydrolyze pGpG to GMP [62,63]. The catalytic mechanism of HD-GYP PDEs is not yet fully understood. However, c-di-GMP hydrolysis appears to involve a metal-activated hydroxide ion that acts as a nucleophile and attacks the phosphorus atom of the ribose phosphate ring [59,63]. Key residues in the active site, including those from the conserved KPG motif and the RXXK motif, help stabilize the substrate (Figure 6). Metal ions further facilitate the reaction by coordinating the oxygen atoms of the ribose phosphate ring and promoting bond cleavage [59,63].

## 3. Development of Cyclic-di-GMP PDEs Inhibitors

The traditional view of c-di-GMP signaling suggests that diguanylate cyclases (DGCs, GGDEF/GGEEF-domain proteins) increase intracellular c-di-GMP levels, promoting biofilm formation, while phosphodiesterases (PDEs, EAL/HD-GYP domain proteins) degrade c-di-GMP, reducing biofilm formation and virulence [64]. This has led to the assumption that inhibiting PDEs would unintentionally enhance biofilm formation, an undesirable effect. However, recent findings reveal a more complex regulation system where overall intracellular c-di-GMP levels alone do not dictate bacterial phenotypes [56,65]. Instead, localized micro-concentrations of c-di-GMP, along with the spatial distribution of regulatory enzymes, adaptor proteins, and binding RNAs, play a crucial role in determining bacterial behavior. The presence of multiple DGCs and PDEs within a single bacterium further contributes to this complexity.

**Figure 7:**
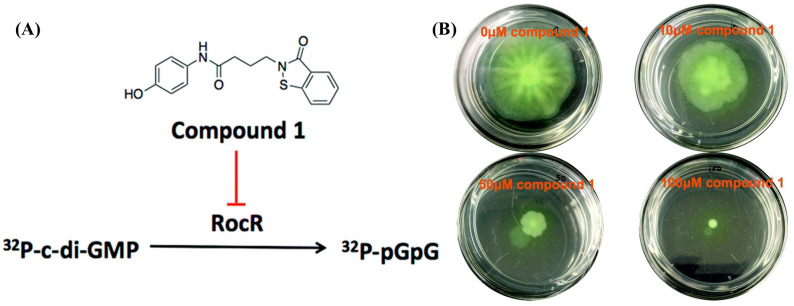
(**A**) Structure of Compound **1** RocR inhibitor; (**B**) Swarming assay showing concentration dependent reduced swarming mobility of PAO1 when treated with Compound **1**. Reproduced from [62].

Studies on PDE mutants and overexpressing clones indicate that certain virulence-associated traits are modulated by multiple PDEs through localized changes in c-di-GMP levels [64,65,66]. Mutants of specific PDE-encoding genes exhibited attenuated virulence in mouse infection models, suggesting that c-di-GMP degradation is highly localized and directly linked to specific regulatory targets [66]. Furthermore, not all c-di-GMP PDEs influence the global c-di-GMP pool, indicating that selective PDE inhibition could have therapeutic applications without broadly altering bacterial signaling. A stark example of this was displayed by the Sintim group, which used computational high-throughput screening to identify a RocR (one of many PDEs in *P. aeruginosa*) inhibitor, Compound **1** (Figure 7A). The inhibitor was able to significantly reduce the swarming ability of the *P. aeruginosa* PAO1 strain (Figure 7B) [65].

## 4. Therapeutic Agents Against *P. aeruginosa* Infections

*P. aeruginosa* is a significant human pathogen, responsible for respiratory tract, urinary tract, wound, and burn infections, as well as colonization of medical devices, leading to hospital-acquired infections [67]. Cystic fibrosis patients are particularly vulnerable to infections caused by this opportunistic bacterium [68]. Given its clinical importance, small molecules that interfere with its signaling pathways could provide valuable insights into *P. aeruginosa* biology and potentially serve as therapeutic agents.

One key regulatory system in *P. aeruginosa* involves c-di-GMP, which controls several bacterial phenotypes. RocR, a major c-di-GMP phosphodiesterase (PDE), plays a crucial role in virulence [64,66]. Hemantha et al. reported that *rocR* gene mutation abolished *P. aeruginosa* virulence in a mouse infection model, suggesting that targeting RocR could be an effective anti-virulence strategy [66]. The Sintim group identified the first small-molecule RocR inhibitor, a benzoisothiazolinone derivative, which selectively inhibited *P. aeruginosa* (PAO1) swarming but not swimming or biofilm formation, further supporting the potential of RocR inhibitors as therapeutic agents against *P. aeruginosa* infections (Figure 7) [65].

## 5. Attenuation of Adherent-Invasive *Escherichia coli* Pathogenicity in Crohn’s Disease

Claret and coworkers have presented data that suggest that c-di-GMP signaling in the adherent invasive *Escherichia coli* (AIEC) pathotype is associated with the development of Crohn’s disease (CD) [69]. CD is a long-term inflammatory disorder that mainly targets the gastrointestinal tract, leading to inflammation that can occur anywhere from the mouth to the anus [69]. CD is classified as an inflammatory bowel disease (IBD), along with ulcerative colitis [70].

Laurent et al. reported that the EAL domain phosphodiesterases yhjH and yahA play a crucial role in regulating the adhesion and invasion of the AIEC strain LF82 by modulating c-di-GMP levels [71]. Their study found that flagella absence in LF82 leads to a loss of type 1 pili, which are essential for intestinal epithelial cell invasion. Mutation of fliA, a key regulator of flagella and pili synthesis, reduces yhjH expression, resulting in elevated c-di-GMP levels, which suppresses type 1 pili production and impairs bacterial adhesion and invasion. Furthermore, they found that overexpression of yhjH or yahA, which degrade c-di-GMP, partially restored type 1 pili synthesis, adhesion, and invasion in the LF82-ΔfliA mutant, demonstrating that lowering c-di-GMP levels counteracts these defects. These findings suggest that reducing c-di-GMP levels in adherent invasive *E. coli* through yhjH and yahA PDE inhibition could attenuate AIEC pathogenicity, providing potential therapeutic insights for Crohn’s disease treatment.

## 6. Potential Anti-Virulence Agents for the Treatment of Cholera Disease

Kolter and colleagues reported that MbaA, an inner membrane protein, plays a key role in the biofilm architecture of *V. cholerae* [72]. Their study showed that mutants lacking MbaA produce an increased amount of extracellular matrix, resulting in thicker biofilms with disrupted structure. This observation underscores the regulatory function of MbaA in biofilm development.

*V. cholerae* is a Gram-negative, facultative anaerobic bacterium and the causative agent of cholera, a severe diarrheal disease. In *V. cholerae*, similar to other organisms, c-di-GMP is involved in the regulation of multiple cellular processes including biofilm formation, motility, and virulence. MbaA and CdgC are GGDEF-EAL domain proteins that function as PDEs in *V. cholerae*, regulating biofilm formation and motility by modulating c-di-GMP levels [72,73].

Though MbaA contains a GGDEF domain, it lacks key residues required for diguanylate cyclase (DGC) activity, reinforcing its role as a PDE [72]. Karatan et al. and Lim et al. demonstrated that MbaA plays a role in gene regulation, specifically by repressing *vps* gene expression in *Vibrio cholerae* [43,74]. The *vps* genes are crucial for producing Vibrio polysaccharide (VPS), a key component of the extracellular matrix that supports the formation of complex biofilm structures. By modulating *vps* gene expression, MbaA influences biofilm development and structural integrity in *V. cholerae*.

Similarly, CdgC, another EAL domain-containing PDE, influences motility and biofilm formation [73]. Tischler and Camilli showed that overexpression of CdgC leads to reduced *vps* gene expression, affecting biofilm matrix production [75]. Additionally, strains overexpressing wild-type CdgC exhibit enhanced motility, whereas a mutated cdgC-AAL allele (disrupting PDE activity) fails to impact motility [43]. These findings underscore the role of MbaA and CdgC in regulating c-di-GMP levels, where mutations or overexpression result in distinct alterations in bacterial biofilm architecture and motility. Consequently, small-molecule inhibitors targeting MbaA, CdgC, or both could serve as potential anti-virulence agents for cholera treatment.

## 7. Potential Anti-Virulence Agents for Black Rot Disease in Plants

The virulence of *X. campestris* is regulated by several factors, including the synthesis of extracellular enzymes and extracellular polysaccharides (EPS), both of which are controlled by the HD-GYP domain regulator RpfG and its cognate sensor kinase RpfC [56]. This two-component signal transduction system perceives and transduces the DSF (diffusible signal factor), whose synthesis is dependent on RpfF [76]. Mutations in *rpfG*, *rpfC*, or *rpfF* reduce virulence, highlighting their role in disease progression [56,77].

*X. campestris* is a Gram-negative, plant-pathogenic bacterium responsible for black rot disease in cruciferous crops such as cabbage, broccoli, and cauliflower. It infects plants through natural openings like hydathodes and stomata or via wounds, spreading systemically through the xylem. Once inside, *X. campestris* produces extracellular polysaccharides (EPS), degradative enzymes, and virulence factors that disrupt water transport, causing wilting, chlorosis, and necrotic V-shaped lesions on leaves [78].

A key breakthrough in understanding *X. campestris* pathogenesis was the demonstration that the HD-GYP domain of RpfG functions as a phosphodiesterase that hydrolyzes c-di-GMP [77]. This finding established a mechanistic link between c-di-GMP signaling and the regulation of virulence, EPS production, and biofilm formation in *X. campestris*. Consequently, targeting RpfG with small-molecule inhibitors presents a promising strategy for developing anti-virulence agents to control black rot disease in economically important crops.

## 8. Targeting c-di-AMP PDEs

Among the second messenger nucleotides, cyclic di-AMP (c-di-AMP) plays a crucial role in cell growth, survival, and virulence regulation, primarily in Gram-positive bacteria [79,80,81]. Its cellular levels are tightly controlled by two enzyme families: diadenylate cyclases (DACs), which synthesize c-di-AMP, and phosphodiesterases (PDEs), which degrade it.

The first c-di-AMP hydrolyzing enzyme (PDE) was identified in Bacillus subtilis [82] and later found in Staphylococcus aureus [83], Listeria monocytogenes [84], and other Streptococcus species [85,86]. PDE enzymes degrade c-di-AMP into phosphoadenyl adenosine (pApA), which is further broken down into AMP. There are four major classes of c-di-AMP PDEs which are GdpP, DhhP, PgpH and CdnP.

## 9. Dhhp-Type Phosphodiesterase

**Figure 8:**
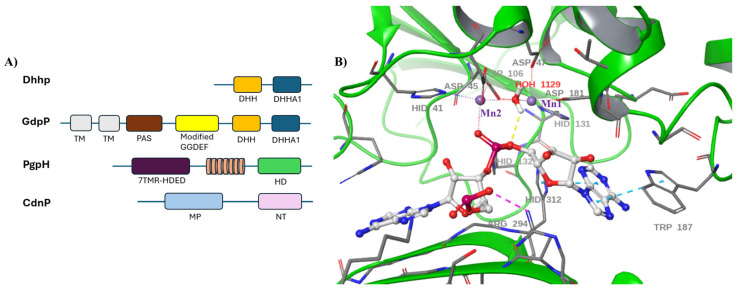
(**A**) Domain structure of c-di-AMP phosphodiesterases [85,86,87,93,94]. (**B**) 3D crystal structure of Rv2837c bound to 5′-pApA (PDB:5JJU) [88].

DhhP-type phosphodiesterases are standalone DHH/DHHA1 domain proteins that degrade c-di-AMP in two steps: first converting it to pApA, then breaking it down into AMP (Figure 8A) [85,87]. The crystal structure of Rv2837c (also called NrnA and CdnP), a standalone DHH/DHHA1 domain protein from *Mycobacterium tuberculosis* (Mtb), bound to 5′-pApA, a hydrolytic intermediate of c-di-AMP, provides valuable insights into the catalytic mechanism of DhhP-type phosphodiesterases (Figure 8B) [88]. The adenine bases of 5′-pApA are positioned perpendicularly, stabilized by π–π interactions with His312. Additionally, His312 forms a hydrogen bond with the phosphate group of the 3′–5′ phosphodiester bond, which is coordinated by two Mn^2+^ ions. This coordination facilitates bond cleavage through a nucleophilic attack by an activated water molecule (Figure 8B). This mechanism is similar to the proposed two-metal catalytic mechanism for EAL PDEs (Figure 4D), and it is likely to be conserved across DHH–DHHA1 PDEs due to their shared binuclear metal center and substrate-binding residues. Standalone DHH/DHHA1 domain proteins have been identified in several bacteria including *B. burgdorferi*, *Mycobacterium* spp., *Mycoplasma pneumoniae*, and *T. maritima* [87,89,90,91,92].

**Figure 9:**
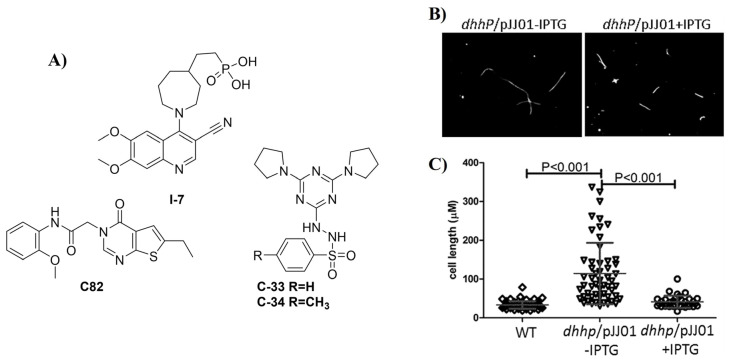
(**A**) Structures of small molecules inhibitors of NrnA discovered by the Sintim group (**C82**) [97], the Bishai group (**C-33** and **C-34**) [99], and Stingray Therapeutics (**I-7**) [98]. (**B**) The DhhP knockout strain of Borrelia burgdorferi shows growth defects; imaged with darkfield microscopy. (**C**) Quantification of cell length from 100 randomly selected cells from (**B**). Reproduced with permission from [83]. Copyright © 2014 American Society for Microbiology-Journals.

Some DhhP enzymes show a preference for linear substrates over cyclic dinucleotides [95,96]. Their essentiality varies across species; in *B. burgdorferi* and *M. pneumoniae*, DhhP is the sole enzyme responsible for c-di-AMP degradation [87,89]. Some homologs also function as nano-RNases, which degrade short RNA oligonucleotides [95]. Meiping Ye et al. [87] found that, unlike in Gram-positive bacteria, DhhP is essential for *B. burgdorferi* growth, and its conditional inactivation did not increase resistance to β-lactam antibiotics, suggesting that c-di-AMP functions may differ across bacterial phyla [87]. Cedric B. et al. further reported that DhhP is critical for potassium homeostasis in *Mycoplasma pneumoniae*, underscoring its role in bacterial adaptation [89]. The *dhhP* mutant in *B. burgdorferi* was defective in OspC production, a major virulence factor, and showed impaired mammalian infection, highlighting the potential of DhhP inhibitors as anti-virulence agents. Additionally, the mutant exhibited elongated cells, suggesting a role for DhhP in cell division (Figure 9B).

Bishai et al. found that NrnA (also called Rv2847c or CdnP) inactivation in Mtb results in the accumulation of extracellular c-di-AMP, triggering a stronger inflammatory response from the host immune system. Due to its role in immune evasion, many research groups have shown interest in developing small-molecule inhibitors targeting NrnA. In 2021, the Sintim group reported **C82** [97], a selective, non-nucleotide-based NrnA inhibitor (Figure 9A). That same year, Srinivas et al. patented a series of phosphonate-based NrnA inhibitors, including **I-7** [98]. More recently, in 2023, Bishai et al. identified another series of NrnA inhibitors, including **C-33** and **C-34** [99] (Figure 9A).

## 10. CdnP-Type Phosphodiesterases

Recently identified in human pathogen *Streptococcus agalactiae*, CdnP phosphodiesterases are cell wall-anchored enzymes that degrade extracellular c-di-AMP [86,93]. CdnP consists of a 5′-nucleotidase domain and a metallophosphoesterase domain, which is essential for its activity (Figure 8A) [100,101]. Bacteria utilize CdnP enzymes to suppress host immunity by cleaving both bacteria-derived c-di-AMP and host-derived 2′,3′-cGAMP. Immune cells recognize bacterial c-di-GMP, c-di-AMP, and host-generated cGAMP, which is synthesized by cGAS upon detecting pathogen DNA, leading to an immune response via the STING-TBK1 pathway [102,103]. The CdnP enzyme, distinct from known bacterial cdN phosphodiesterases, functions in tandem with the ectonucleotidase NudP to sequentially break down extracellular c-di-AMP into adenosine [104]. Currently, no structural characterization is available for CdnP PDE.

## 11. PgpH and GdpP-Type Phosphodiesterases

**Figure 10:**
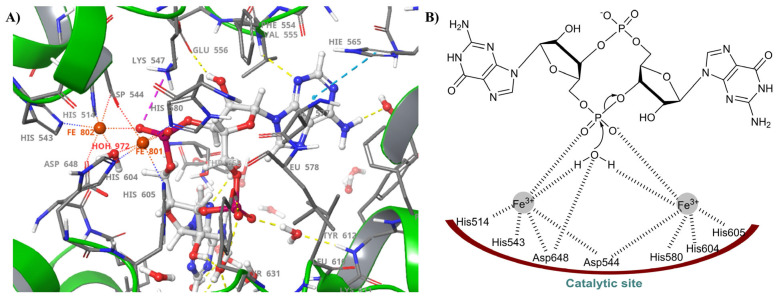
(**A**) The crystal structure of the PgpH HD (PDB: 4S1B) domain complexed with c-di-AMP reveals detailed molecular interactions. A bridging water molecule (HOH972) is positioned between the two metal ions [94]. (**B**) Proposed catalytic mechanism for PgpH HD domain PDE [94].

PgpH-type phosphodiesterases were first identified in *Listeria monocytogenes* and are widespread among Firmicutes, though absent in *Staphylococcus* and *Streptococcus* species [81,84,105]. PgpH contains eight transmembrane helices, an extracellular seven transmembrane helix-HDED domain (7TMR-HDED) domain, seven transmembrane helices, and a cytosolic HD phosphohydrolase domain, which hydrolyzes c-di-AMP into pApA (Figure 8A) [94]. Crystal Structure of *L. monocytogenes* PDE PgpH HD domain in complex with Cyclic-di-AMP give insight into the catalytic mechanisms of PgpH PDE (Figure 10). Structural analysis reveals that in the Fe-bound active site, a bridging water molecule is positioned beneath the 5′ phosphate of nucleotide, the target of hydrolysis. This water is activated by the two metal ions and a hydrogen bond with Asp648, which also coordinates Fe1. The water/hydroxide then acts as a nucleophile, attacking the phosphorus atom, resulting in the release of the 3′ hydroxyl of nucleotide 2. The oxyanion formed is stabilized by the ammonium group of Lys547, which, along with a solvent water molecule, facilitates protonation to complete the reaction (Figure 10B). Given the conserved nature of the HD domain, this catalytic mechanism is likely shared among other HD-domain phosphodiesterases.

The GdpP-type phosphodiesterase was first identified in *Lactococcus lactis* and later found in various Firmicutes, including *B. subtilis*, *S. aureus*, *S. pneumoniae*, and *Enterococcus* species [77,79,82,83,85,106,107,108,109]. GdpP contains transmembrane helices, a PAS domain, a degenerated GGDEF domain and a DHH/DHHA1 phosphodiesterase domain, which degrades c-di-AMP into pApA (Figure 8A) [105,110]. The catalytic mechanism of GdpP PDEs are the same with DhhP since they both contain DHH/DHH1 domains [111]. GdpP is regulated by heme and nitric oxide (NO), which inhibit its activity, as well as the bacterial stress signal (p)ppGpp, which competitively inhibits its catalytic function [109,112]. In many Firmicutes, GdpP inactivation leads to elevated c-di-AMP levels, resulting in cell wall-damaging antibiotics resistance and altered virulence [81,112]. However, studies by Jan Gundlach et al. have demonstrated that a *gdpP pgpH* double mutant is toxic to *Bacillus subtilis*, prompting the inactivation of diadenylate cyclase CdaA to counterbalance c-di-AMP accumulation [81]. Similarly, research by TuAnh N. H. et al. [94] found that a *gdpP pgpH* double mutant in *Listeria monocytogenes* exhibited enhanced host inflammation, intracellular growth defects, and significantly reduced virulence in a murine infection model, suggesting that dual inhibitors of GdpP and PgpH could serve as anti-virulence agents against Listeria infection.

## 12. Bacterial Anti-Phage Defenses

Bacteria respond to invading genetic elements in primarily four different ways. First, is through CRISPR [113] (clustered regularly interspaced short palindromic repeats), second is through CBASS [114] (Cyclic oligonucleotide-Based Anti-phage Signaling System), third through Pycsar [115] (pyrimidine cyclase system for antiphage resistance), and fourth through Thoeris antiphage defenses. Although Pycsar signaling is also an exciting area of interest, this signaling utilizes cyclic-mononucleotides and so will not be discussed, but the reader is referred to an excellent review on this topic by Hobbs and Kranzusch [115].

CRISPR systems are extremely well explored, as the CRISPR-Cas9 system is widely used to insert desired engineered genetic elements into organisms of interest [116]. The CRISPR system is, in general, an adaptive immune system that bacteria use to identify known phages and launch a variety of anti-phage defenses which ultimately lead to viral clearance, cell dormancy, or cell death. Some Cas proteins utilize cONs (specifically c-hexa-AMP or c-tetra-AMP) to act as an ‘on’ signal for these responses, and thus clearance of the second messenger is crucial to turn ‘off’ the signal once viral clearance has occurred. Similarly to the cyclic dinucleotide signaling present in bacteria, specific PDEs recognize and open the ring of the cONs [117,118,119].

Because bacterial resistance is considered a hurdle to the development of phage antibacterial drugs [120,121,122,123], we also propose that a c-hexa-AMP or c-tetra-AMP PDE inhibitor could potentially be combined with phage therapy. In such combination therapy, the cON PDE inhibitor could prevent resistance to such phage therapy [124,125].

**Figure 11:**
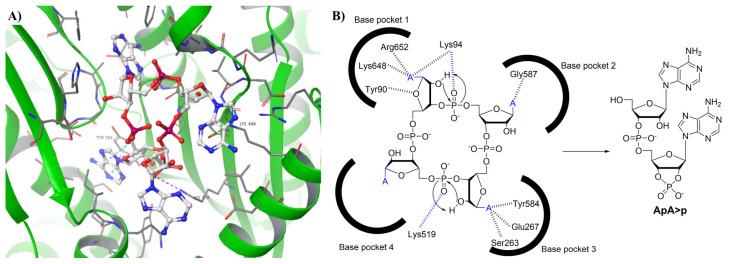
(**A**) Csm1 protein bound to cA_4_ (PDB: 6O7H). The residues interacting with the ligand are labeled [133]. (**B**) The proposed mechanism of cA_4_ hydrolysis by Athukoralage J. S. et al. [117] The 2′-OH is the nucleophilic group responsible for ring opening. The interactions highlighted in blue were not indicated in the crystal structure but were both within 3.7 Å when measured. The proposed hydrolysis mechanism of cA_6_ is identical to cA_4_ except for 2 additional pockets for all 6 nucleotide bases to bind.

**Figure 12:**
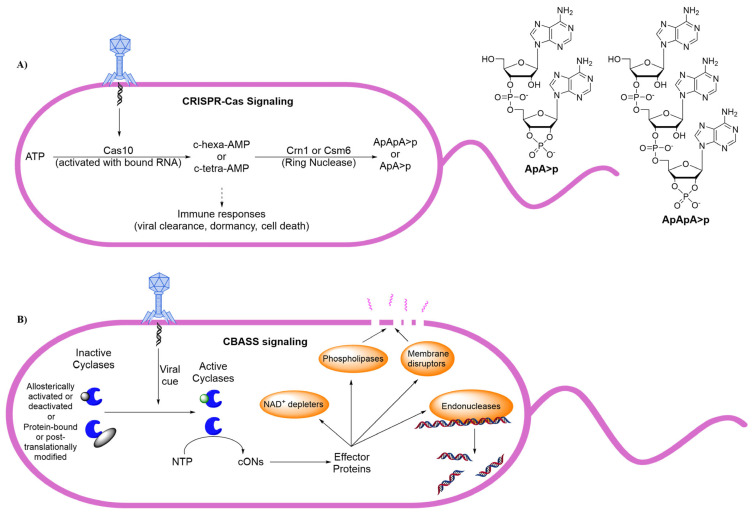
(**A**) A high-level overview of CRISPR signal cascade in bacteria. (**B**) An overview of CBASS signaling in bacteria which is used to combat phage propagation through abortive infection.

Csm1 (also called Crn1) is a ring nuclease that can cleave c-tetra-AMP and Csm6 is a ring nuclease which is capable of cleaving c-hexa-AMP (Figure 11) [117,126]. The most interesting part of these hydrolysis mechanisms is that neither nucleophilic water nor a protein residue is responsible for cleaving the phosphate diester bond. Instead, it is the neighboring 2′OH on the ribose sugar which ultimately results in a new 2′,3′-cyclic monophosphate. This mechanism happens twice to result in two identical fragments which are half the size of the original ring. The nomenclature for this product is not extremely well-established, although some leading authors describe these oligonucleotides as 5′-ApA>p and 5′-ApApA>p with the carrot denoting the 2′,3′-cyclic phosphate (See Figure 12) [117].

CBASS and Thoeris signaling is less well understood than CRISPR-Cas systems, although our understanding is quickly expanding. While CRISPR is more akin to an adaptive immune system, CBASS and Thoeris are likened to innate immune systems. cON cyclases are kept in an inactive state by either post-translational modifications or allosteric activation. The viral cue, which activates the cON cyclases, has yet to be fully elucidated but it has been shown that at least one CBASS cyclase senses viral RNA to initiate immunity [127] and when studying dsDNA phages it has been shown that mutations in the phage prohead protease can result in CBASS subversion [128]. Additionally, similarity to eukaryotic systems has also resulted in the hypothesis that some CBASS cyclases are activated by short peptides [129]. While the underlying mechanics of cyclase activation are not fully understood, the result is ultimately the production of cONs such as 2′,3′,3′-c-tri-AMP, 3′,3′,3′-c-tri-AMP, 3′,3′-cGAMP, and 3′,3′-c-di-UMP in the case of CBASS and cADPR in the case of Thoeris [115,130]. The cON then binds to effector proteins which elicits cell death through lysis, genome destruction, ion release, or NAD^+^ depletion (Figure 12B). We have found no reports of bacterial PDEs responsible for degrading these cONs although a recent discovery has shown that some phages encode proteins capable of both sequestering and hydrolyzing both CBASS-specific and Theoris-specific cONs [115,131,132]. Since this abortive infection mechanism ultimately halts phage propagation, it could be a powerful tool for the development of novel antibacterials if exploited in pathogenic bacteria.

While the idea of programmed cell death may seem counterintuitive for viral clearance, it is ultimately for the benefit of the entire population for a single bacterium to die in order to halt phage propagation. This abortive infection mechanism is so highly conserved that there is evidence for ~36% of all bacteria to have at least one CBASS, Pycsar, Thoeris, or CRISPR antiphage defense system [115].

## 13. Human cON PDEs as Potential Drug Targets

**Figure 13:**
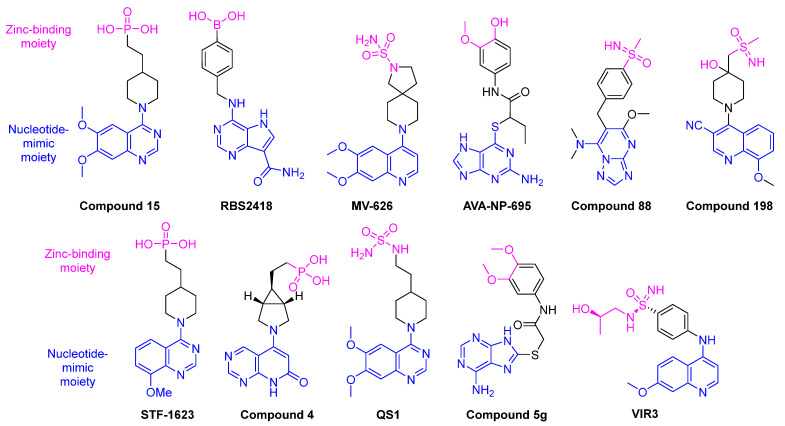
Structures of select potent ENPP1 inhibitors currently under investigation for clinical usefulness. These inhibitors all competitively bind to ENPP1 by interacting with one of the two nucleotide-binding pockets and interacting with the Zn^2+^ cofactor(s) [142]. Compounds were selected to include those that inhibited ENPP1 with single-digit nanomolar or picomolar inhibitory concentrations (K_i_) as well as additional compounds with higher K_i_ values, but representative of structurally different compound classes.

Human antimicrobial defense mechanisms, including the production of type I interferons (IFNs), are triggered by microbial DNA from bacteria, viruses, and potentially parasites through the cytosolic sensor known as STING. STING signaling is activated by cONs produced by intracellular bacteria or synthesized by the enzyme cGAS in response to cytosolic self-DNA or microbial DNA [32]. ENPP1, the phosphodiesterase that hydrolyzes cONs such as 2′,3′-cGAMP to AMP and GMP, plays a critical role in regulating this pathway. However, ENPP1 is often upregulated in tumor microenvironments, contributing to immunosuppression (see Figure 2B) [134]. Targeting ENPP1 to prevent cON degradation has emerged as a promising strategy to enhance immune activation against both pathogens and tumors. There are currently three ENPP1 inhibitors which have begun undergoing clinical trials (RBS2418, Figure 13 [135,136], and SR-8541A [137], and TXN10128 [138]) for patients with advanced or metastatic solid tumors. The structures of SR-8541A and TNX10128 have not been disclosed. A phase 2 trial is also currently recruiting for SR-8541A for combination therapy with Botensilimab [139]. At the time of writing, there are no outcomes posted on clinicaltrials.gov for any of the clinical trials for these ENPP1 inhibitors.

**Figure 14:**
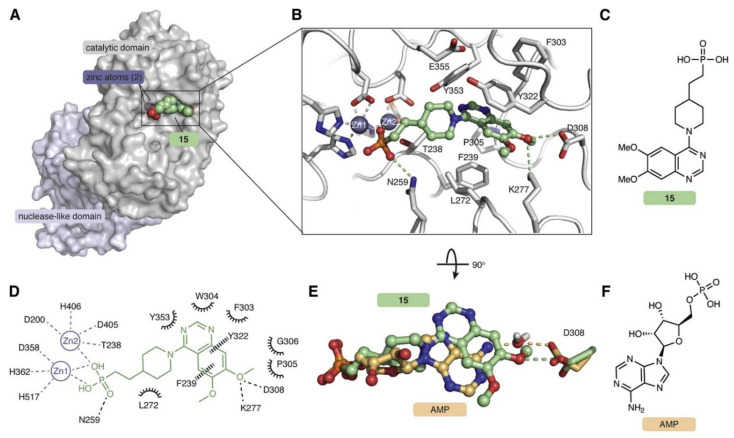
Docking of Compound **15** shows competitive binding with interactions between the phosphate and zinc cofactors as well as K277 and D308 which are important for binding to the nucleotide base. Reproduced with permission from [140]. © 2020 Elsevier.

A plethora of other ENPP1 inhibitors have been discovered in recent years with IC_50_ values ranging from low micro-molar to pico-molar. While other reviews attempt to categorize the known ENPP1 inhibitors to probe for structural similarities [134,140,141,142,143], we will instead focus on more clinically advanced candidates such as RBS2418, or compounds that inhibited ENPP1 with single-digit nanomolar or picomolar inhibitory concentrations (K_i_) as well as additional compounds with higher K_i_ values, but representative of structurally different compound classes. Compound **15** was also chosen as a representative ENPP1 inhibitor as the binding mode to the enzyme has been solved (Figure 14). Recently, Wells and co-workers reported interesting VH domains that allosterically inhibit ENPP1 [144].

**Figure 15:**
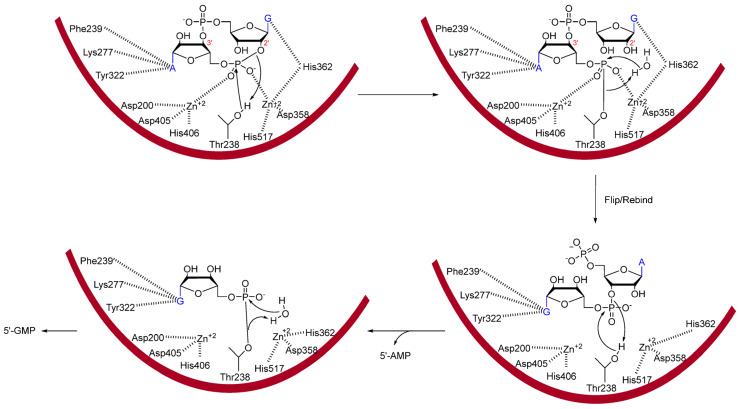
Mechanism of ENPP1 hydrolysis of 2′,3′-cGAMP. ENPP1 first hydrolyses the G-2′-phosphate linkage resulting in 5′-pApG. The dinucleotide is then reoriented for further hydrolysis to AMP and GMP. Residues forming interactions were taken from PDB: 6AEK [145].

The active site of ENPP1 anchors both the nucleotide bases as well as one of the two phosphate groups utilizing several residues (Figure 15) [145]. First, Phe, Lys, and Tyr side chains form interactions with the adenosine base and His362 forms an interaction with the guanosine base. This orients the G-2′phosphate group towards two Zn^2+^ ions which are anchored in the active site by several conserved Asp and His residues. The oxygen atom on Thr238 will ultimately become the nucleophilic atom responsible for breaking the G-2′-phosphoester bond. Then, surrounding water will release the linear substrate from Thr238. After the first hydrolysis, the linear dinucleotide (5′-pApG-3′) can then rebind in the opposite orientation with the guanosine base occupying the site which was previously occupied by the adenine base. Hydrolysis occurs again utilizing the same mechanism, ultimately resulting in 5′-AMP and 5′-GMP.

Clinically advanced inhibitors of ENPP1 are all structurally related, having both nucleotide-mimicking and zinc-binding moieties (Figure 13). Compound **15** is used as an example in Figure 14 to depict the binding mode [146].

## 14. Disappearance of cONs in Fungi

Thus far, cONs have been observed in every branch of the tree of life, from bacteria and archaea to plants and animals. However, little can be said for the role or even presence of cONs in fungi. A 2017 article highlights the effect of bacterial cONs on the fungus *Saccharomyces cerevisiae* [147]. The authors note that induced c-di-GMP production causes significant growth reduction to the studied yeast, although their control experiments show little of native cONs present in the fungal cells. Because bacterial and human concentrations of cONs are in the low- to mid-µM range, the fact that no cONs were present in this concentration indicates that fungi may not use this type of second messenger at all. However, more information is needed in this area of the field before adequately drawing conclusions.

## 15. Future Prospects

Although the field of cON research has exploded over the past decades, research has primarily focused on human cGAS-STING signaling and bacterial c-di-GMP cyclases (for biofilm inhibition). c-di-AMP and 3′,3′-cGAMP PDEs are still in an area which is underutilized in drug discovery. Specifically, a plethora of research on the NrnA enzyme (also called CdnP or Rv2837c) has highlighted its potential as an anti-TB drug target. Additionally, understanding and then hijacking the bacterial CBASS pathway may lead to a new class of antibacterials which cause bacterial self-killing. Ultimately, the goal of this review is to reinvigorate the field with the exciting potential of these cON PDEs as druggable targets in humans and bacteria.

## 16. Conclusions

**Figure 16:**
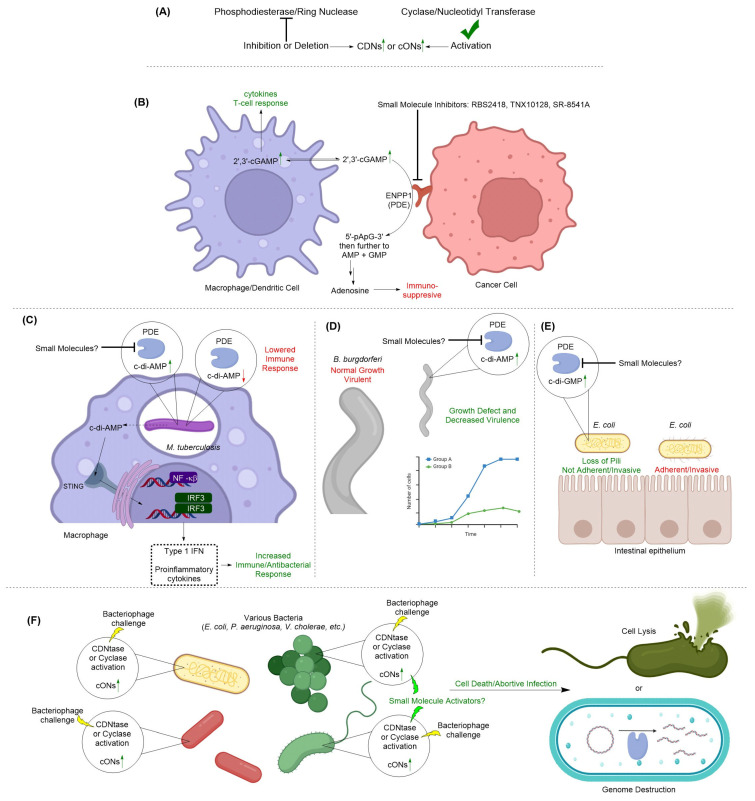
(**A**) A general schematic of how CDN or cON accumulation can occur in cells is shown. (**B**) ENPP1 inhibitors currently in clinical trials are used for cancer immunotherapy as ENPP1 overexpression can lead to immunosuppression. (**C**) Inhibition of NrnA (PDE) in *M. tuberculosis* leads to increased host immune response and antibacterial activity. (**D**) Inhibition of DhhP (PDE) in *B. burgdorferi* leads to decreased virulence and growth defect phenotype. (**E**) Inhibition of *E. coli* c-di-GMP PDE leads to loss of type 1 pili and loss of adherent and invasive phenotype. (**F**) During phage infection, CBASS activation leads to production of cONs, which results in abortive infection through lysis or genome destruction. There are no known bacterial PDEs that degrade CBASS-related cONs.

cON PDEs represent a new class of exciting drug targets (Figure 16). Antibacterial discovery has ground to a halt for several decades, and new antibacterial compounds with novel mechanisms of action are needed urgently. Although various groups are interested in these targets in bacteria to disperse biofilms and decrease virulence, more research is needed to identify novel chemotypes that inhibit bacterial PDEs. Conversely, we are approaching a new era of cancer immunotherapy with the ongoing clinical trials of ENPP1 inhibitors. However, nucleotide analogs alone may not be enough to target a wide range of cancers, keep resistance generation low, or reduce side effects associated with off-target interactions. Thus, novel scaffolds that inhibit human cON PDEs are also needed.
